# Recent Advances in Gluten-Related Disorders

**DOI:** 10.3390/ijms26041749

**Published:** 2025-02-18

**Authors:** Sylwia Smolinska, Krzysztof Jurkiewicz, Emilia Majsiak

**Affiliations:** 1 Department of Clinical Immunology, Faculty of Medicine, Wroclaw Medical University, Parkowa 34, 51-616 Wroclaw, Poland; krzysztof.jurkiewicz@student.umw.edu.pl; 2 Department of Health Promotion, Faculty Health of Sciences, Medical University of Lublin, Staszica 4/6, 20-081 Lublin, Poland; emiliamajsiak@umlub.pl

## 1. Introduction

The articles featured in this Special Issue of the *International Journal of Molecular Sciences*, entitled “Recent Advances in Gluten-Related Disorders”, present significant advancements in the understanding of gluten hypersensitivity, offering valuable insights into mechanisms, diagnostics, and potential therapeutic strategies. Gluten is the main storage protein of wheat grains and a complex mixture of hundreds of related but distinct proteins, mainly gliadin and glutenin. Similar storage proteins, such as secalin in rye, hordein in barley, and avenins in oats, are collectively referred to as “gluten”. Nowadays, the consumption of gluten-free products is becoming an increased dietary habit in the general population. An increased number of people are beginning to erroneously view gluten as a toxic compound, an idea popularized by people without medical or scientific backgrounds who adopt gluten-free diets. The suggestion that the avoidance of gluten improves health is medically unjustified. The aim of this SI is to promote active debate in the scientific community about gluten to show the importance of gluten intake as well as the only valid medical reasons for its avoidance. In this Special Issue, we have collated papers describing the most recent advances in the immunopathology of gluten digestion, the diagnostic biomarkers of gluten-related diseases, the susceptibility of gluten-related disorders, and new potential therapies for the treatment of gluten-related disorders, as well as the importance of gluten intake in the daily diet. Collectively, these studies underscore the importance of combining experimental and computational approaches to tackle complex immunological and molecular challenges, particularly in the context of gluten hypersensitivity and IgE-mediated wheat allergies.

## 2. The Intersection of Mechanisms and Diagnostics

Preda et al. present a comprehensive review of IgE-mediated wheat allergies, detailing their diverse phenotypes, ranging from food-induced anaphylaxis to occupational asthma in bakers. Their work highlights the centrality of allergen-specific IgE antibodies to wheat proteins, such as omega-5 gliadin (Tri a 19) and lipid transfer proteins (Tri a 14), in diagnostic strategies. The authors emphasize the role of multiplex immunoassays and basophil activation tests (BATs) as emerging tools for more precise diagnostics. Notably, this study underscores the significance of molecular allergology in differentiating between genuine sensitizations and cross-reactive responses, particularly in patients with coexisting grass pollen allergies [1]. Such findings align with the broader goal of integrating molecular diagnostics into routine clinical practice to enhance specificity and reduce false-positive outcomes.

Complementing these insights, Srisuwatchari et al. highlight the diagnostic utility of epitope-specific IgE, IgG4, and IgG1 antibodies, demonstrating their potential to distinguish between clinical wheat allergies and mere sensitization, thus refining diagnostic accuracy [2]. Moreover, Ogino et al. identified peroxidase-1 and beta-glucosidase as cross-reactive wheat allergens in individuals with grass pollen-related wheat allergies, further emphasizing the importance of molecular diagnostics in clarifying complex sensitization patterns [3]. Pinhal et al. contribute to this growing body of evidence by reporting new wheat allergens identified in cases of undercooked wheat allergy, illustrating how food processing methods can influence allergenic potential [4].

Together, these findings reinforce the pivotal role of molecular allergology in clinical practice, offering tools to enhance specificity, reduce false-positive outcomes, and ultimately improve patient management strategies for wheat allergies.

## 3. Innovations in Dietary-Based Therapeutics

Jorgensen et al. focus on dietary-based therapeutic approaches for gluten hypersensitivity, an IgE-mediated disorder that poses substantial dietary and clinical challenges. Their review identifies five promising therapeutic strategies, including the enzymatic hydrolysis of gluten, fermentation, and the genetic modification of wheat proteins. Two of these approaches have progressed to limited human clinical trials, showcasing their translational potential. The authors also provide a detailed analysis of preclinical animal studies, which have demonstrated the efficacy of reducing gluten allergenicity through thioredoxin treatment and the administration of heat-killed *Listeria monocytogenes*. These findings illuminate pathways for mitigating gluten hypersensitivity without relying solely on strict gluten avoidance, a paradigm shift that could significantly improve quality of life for affected individuals [5].

Gao et al. and other authors critically evaluate processing methods to create hypoallergenic wheat products, discussing the potential and limitations of techniques such as enzymatic hydrolysis and fermentation in reducing wheat allergenicity [6,7]. In addition, Cabanillas provides a comprehensive overview of gluten-related disorders, including celiac disease, wheat allergy and non-celiac gluten hypersensitivity, highlighting the distinct pathophysiological mechanisms and the need for tailored therapeutic approaches for each condition [8]. In addition, Revyakina et al. evaluate the tolerability of a new gluten-free cereal snack in children with gluten food allergy, suggesting potential dietary alternatives that could reduce the burden of strict gluten avoidance. Together, these studies, as well as those conducted by other teams, underscore the importance of developing and validating dietary interventions that go beyond strict gluten avoidance, with the goal of improving the quality of life for people with gluten sensitivity [9,10,11].

## 4. Advancing Oral Therapies for Celiac Disease

In their groundbreaking study, Liu et al. investigate a novel enzyme-based approach to celiac disease, an autoimmune condition triggered by the ingestion of gluten peptides resistant to enzymatic breakdown in the digestive tract. The authors focus on Bga1903, a glutenase derived from the Gram-negative bacterium *Burkholderia gladioli*. Structural and enzymatic analyses of Bga1903 revealed its moderate ability to degrade pro-immunogenic gluten peptides. By employing site-directed mutagenesis, the team successfully enhanced the enzyme’s substrate specificity toward key gluten motifs such as QPQ, producing a double-site mutant, E380Q/S387L, with a 34-fold increase in its specificity constant. This enhanced specificity allows the enzyme to more effectively break down toxic gluten peptides, presenting a promising candidate for oral therapy in celiac disease management. Liu’s work exemplifies the innovative use of structural biology to address critical gaps in the therapeutic landscape for autoimmune disorders [12].

Recent advancements in enzyme therapy for celiac disease further support these findings. Wei et al. review the use of gluten-degrading enzymes such as prolyl endopeptidases, cysteine proteases, and subtilisins, which cleave proline- and glutamine-rich gluten peptides. These enzymes show promise as oral therapies for CeD, offering critical support for patients with high gluten sensitivity. Innovative strategies, including enteric coating and enzyme modification, are highlighted as essential for ensuring stability and efficacy in the digestive tract [13].

Similarly, Pultz et al. explore a range of gluten-degrading enzymes, emphasizing their potential to neutralize immunogenic gluten peptides under gastro-duodenal conditions. Their work highlights the importance of enzyme stability and activity across varying pH levels, further advancing the development of safe and effective CeD therapies [14].

## 5. Expanding Diagnostic Frontiers in WALDA

Faihs et al. investigate Wheat Allergy Dependent on Augmentation Factors (WALDA), a condition that exemplifies the interplay between allergenic proteins and external cofactors, such as exercise, alcohol, or nonsteroidal anti-inflammatory drugs. Using advanced cellular in vitro tests, including BAT and basophil histamine-release assays, the authors reveal distinct sensitization profiles in patients. Their study identifies alcohol-free wheat beer and hydrolyzed wheat proteins as novel test substances with diagnostic potential, thereby expanding the toolkit for evaluating complex sensitization patterns in WALDA. Importantly, their findings demonstrate the utility of combining cellular assays with traditional immunological methods to achieve a more nuanced understanding of allergenic responses.

The information on the latest scientific advances in the field can be expanded in this aspect as well. Gabler et al. study basophil activation in response to both gluten and non-gluten proteins in patients with wheat-dependent exercise-induced anaphylaxis (WDEIA), highlighting the complex sensitization profiles that can affect diagnostic approaches. Other authors provide a comprehensive overview of WDEIA, discussing its subtypes, diagnostic challenges and management strategies, thus contributing to a more nuanced understanding of this condition [15,16,17,18].

By analyzing these studies together, one can observe a clear emphasis on the importance of integrating advanced cellular assays with traditional immunological methods to improve diagnostic accuracy and deepen our understanding of allergic reactions in conditions, such as WALDA and WDEIA.

## 6. Towards a Holistic Understanding of Wheat Allergy

This Special Issue, with its focus on molecular advances in food allergy research, highlights the power of interdisciplinary approaches that merge experimental and computational methodologies. The articles featured here emphasize the critical need for precise molecular diagnostics, innovative therapeutic strategies, and advanced modeling to address the multifaceted challenges posed by gluten hypersensitivity. For instance, the combination of structural analyses with immunological assays, as demonstrated in these studies, provides a more comprehensive view of allergen–host interactions at both molecular and systemic levels.

Additionally, the broader implications of these findings extend beyond wheat allergies. The methodologies and insights presented here are applicable to other allergenic systems, particularly those involving complex protein interactions and immune dysregulation. For example, the identification of leucine-rich repeat-containing protein 15 (LRRC15) as a potential modulator of lung fibrosis in post-COVID-19 complications opens new avenues for exploring similar pathways in allergenic diseases.

## 7. Challenges and Future Directions

Despite these advances, significant challenges remain. The variability in allergen profiles among individuals necessitates personalized diagnostic and therapeutic approaches. Furthermore, the transition from preclinical models to clinical applications requires robust validation in diverse patient populations. Addressing these challenges will demand continued collaboration across disciplines, integrating insights from molecular biology, immunology, and computational modeling.

## 8. Conclusions

This Special Issue exemplifies the transformative potential of interdisciplinary research in advancing our understanding of food hypersensitivities. By leveraging experimental rigor and computational innovation, these studies pave the way for more effective diagnostics and therapies, ultimately improving patient outcomes. As the field continues to evolve, it is imperative to maintain a holistic perspective, recognizing the interconnectedness of molecular, cellular, and systemic factors in allergenic diseases. Additionally, the study shows that only proper and precise diagnostics can lead to the correct decision regarding the elimination of gluten from the diet.
